# Performance Evaluation of SARS-CoV-2 Viral Transport Medium Produced by Bangladesh Reference Institute for Chemical Measurements

**DOI:** 10.3390/diagnostics13091622

**Published:** 2023-05-04

**Authors:** Mamudul Hasan Razu, Bayzid Bin Monir, Md. Moniruzzaman, Sawgotom Sarkar, Sonia Akhter, Sabiha Kamal, Md. Abu Hasan, Mirola Afroze, Khandaker Md. Sharif Uddin Imam, Mala Khan

**Affiliations:** 1 Bangladesh Reference Institute for Chemical Measurements (BRICM), Dr. Qudrat-E-Khuda Road, Dhanmondi, Dhaka 1205, Bangladesh; 2 National Institute of Laboratory Medicine and Referral Center (NILMRC), Sher-E-Bangla Nagar, Agargoan, Dhaka 1207, Bangladesh

**Keywords:** coronavirus disease 2019 (COVID-19), Hank’s balanced salt solution (HBSS), *N* gene, rRT-PCR, viral transport medium (VTM)

## Abstract

A viral transport medium (VTM) was developed following the Centers for Disease Control and Prevention, USA (US-CDC) standard operating procedure (SOP) DSR-052-05 with necessary improvisation and was used for storing coronavirus disease 2019 (COVID-19) swab specimens. Considering Bangladesh’s supply chain and storage conditions, improvisation was essential for extending sample storage time while retaining efficiency. In-house VTM was produced using Hank’s balanced salt solution (HBSS) supplemented with 1% bovine serum albumin V (BSA), 0.5 µg /mL of gentamicin sulfate, and 100 µg/mL of fluconazole. The produced VTM composition, quality, sterility, specificity, and efficiency were verified in-house and through an independent contract research organization (CRO). An accelerated stability study projected that under the recommended temperature (4 °C), it would remain stable for four months and preserve samples for over a month. The real-time reverse transcriptase–polymerase chain reaction (rRT-PCR) test detected the targeted *N* gene and *ORF1ab* gene from the VTM stored samples. Our VTM is equally as effective as the Sansure Biotech VTM in keeping SARS-CoV-2 RNA specimens detectable in rRT-PCR (100% sensitivity and specificity in random and blinded samples). In conclusion, the BRiCM VTM will make the battle against pandemics easier by effectively collecting and storing nasopharyngeal and oropharyngeal swabs for COVID-19 detection.

## 1. Introduction

The coronavirus disease 2019 (COVID-19), emerging as a killer threat to humanity, has become the center of attention of the world community regarding the health and security of people. It is caused by the severe acute respiratory syndrome coronavirus 2 (SARS-CoV-2). Since its detection as a pneumonia of initially unknown etiology in Wuhan City, Hubei Province of China, on 31 December 2019, it has caused more than 305 million cases and 5 million deaths worldwide, with more than 1.5 million cases and more than 28 thousand deaths in Bangladesh [1,2]. This enduring and rapid rampage of SARS-CoV-2 has led to a worldwide public health emergency [3,4]. The World Health Organization (WHO) declared SARS-CoV-2 a pandemic on 11 March 2020 [5]. To control the outbreak and rapid spread of the COVID-19 pandemic, it is clear that enormous and rapid initiatives for testing, contact tracing, and data collection to predict probable hotspot areas are necessary [6,7]. The current diagnosis methods of COVID-19 are associated with the detection of the virus using genomic techniques using either drop sequencing or polymerase chain reaction (PCR)–based methods [8,9,10]. The WHO has recommended nucleic acid testing (NAAT) of the upper respiratory tract (URT) and lower respiratory tract (LRT) specimens as the method of choice for detecting SARS-CoV-2 [7,11]. Real-time reverse transcriptase-polymerase chain reaction (rRT-PCR), typically performed from URT specimens, is considered the gold standard for detecting SARS-CoV-2 [8]. Nasopharyngeal (NP) and oropharyngeal (OP) swabs are the two main recommended types of URT specimens for COVID-19 rRT-PCR diagnostic testing [12,13,14]. The most preferred specimen is an NP swab carried in a specific viral transport medium (VTM) to a clinical molecular laboratory (CML), where flocked nylon swabs are standard for collecting NP specimens [15].

Many developing countries, including Bangladesh, rely on imported tools and equipment to fight SARS-CoV-2. To contain the virus spreading, border and international transit lockdowns were initial and lasting responses for most of the country. It has created a supply chain bottleneck, hampering essential healthcare services and other sectors. Esbin et al. [16] reviewed alternative SARS-CoV-2 nucleic acid detection methods to bypass this bottleneck, but all methods start with proper sample collection tools and techniques. As the COVID-19 pandemic prevails with the frequent emergence of new variants, mass PCR testing in CML faced severe challenges in maintaining adequate supplies of VTM [17]. In addition, the pandemic has enabled worldwide research laboratories and medical consortiums to unveil clinical characterizations, epidemiology, and causes of COVID-19 [9,18,19,20,21]. One of the sustainable ways to come out of the situation is to develop domestic alternatives for much-needed healthcare tools.

To empower our national SARS-CoV-2 detection capacity and meet the growing demand for COVID-19 tests, the Bangladesh Reference Institute for Chemical Measurements (BRiCM), Ministry of Science and Technology (MoST), The People’s Republic of Bangladesh took the initiative to manufacture a VTM for collecting NP or OP swabs. The primary purpose of VTM is to collect and preserve viruses for amplification using NAAT and virus culture. Different formulations for VTM exist, mainly consisting of a buffered salt solution, a complex source of protein or amino acids, and antimicrobial agents. Though more straightforward in formulation, scientists used complex media, such as Copan’s universal transport medium (UTM) and VTM, while developing NAAT assays for detecting respiratory pathogens [17]. Here, at the BRiCM, we intend to formulate and manufacture an efficient, cost-effective, and reliable VTM kit, according to the Centers for Disease Prevention and Control, USA (US-CDC) guidelines and formulation with modifications to meet demand. To the best of our knowledge, it is the first attempt to develop such transport media in Bangladesh. This article reports the formulation, evaluation, and calibration of a cost-effective, easily producible, and readily available VTM solution developed at the laboratory of the BRiCM to support the diagnosis process and control of COVID-19 in Bangladesh.

## 2. Materials and Methods

### 2.1. Preparation of VTM

**Figure 1:**
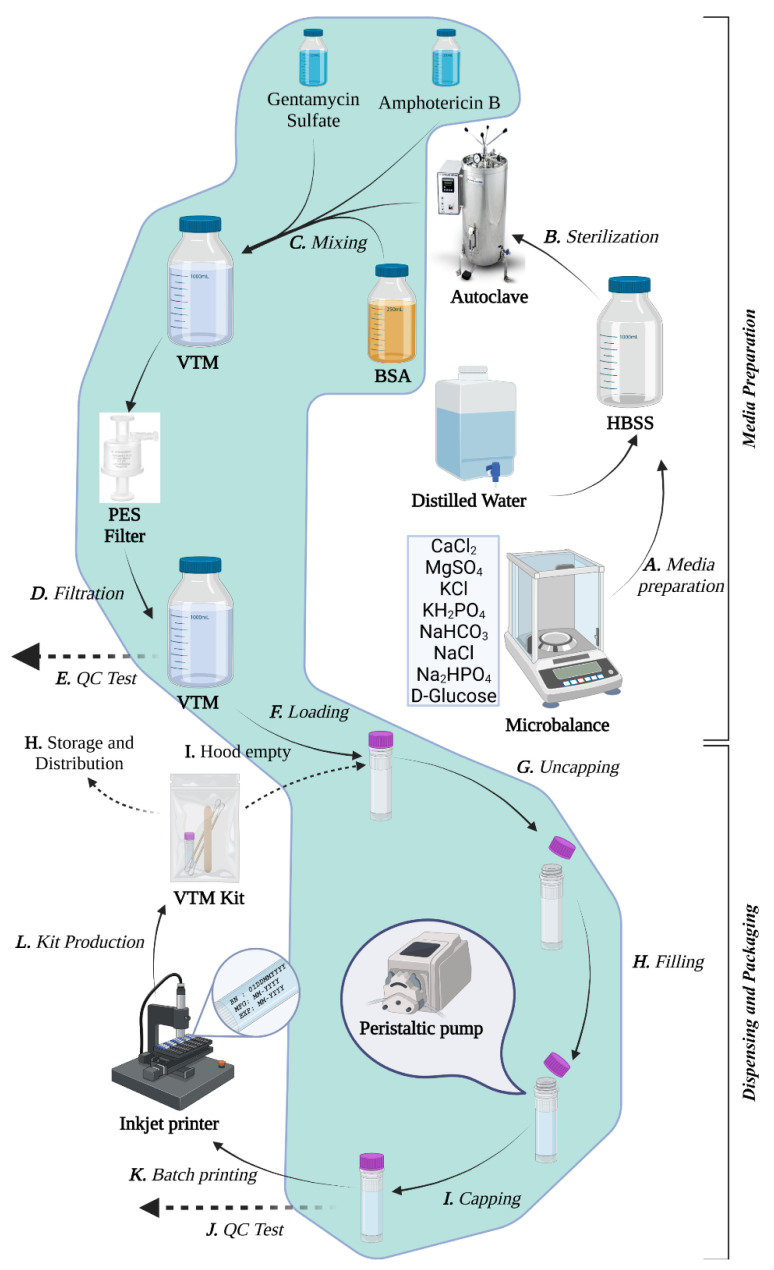
Schematic diagram of BRiCM VTM production workflow. The figure shows the steps in a continuous workflow diagram of the VTM preparation and packaging steps (italicized with bold numbering). Processes in the green-shaded area were performed in the sterile cabinet.

VTM was prepared using modified US-CDC standard operating procedure (SOP) DSR-052-05 [22] with Hank’s balanced salt solution (HBSS) (as a balanced salt solution). In this step, the mixture was sterilized by autoclaving at 121 °C for 30 min. In total, 0.5 µg/mL of gentamicin sulfate (CAS number: 1405-41-0, Merck KGaA, Darmstadt, Germany) was used as an antibacterial. Instead of recommended amphotericin B as an antifungal, 100 µg/mL fluconazole (CAS No.: 86386-73-4) was used as both possess fungicidal properties [23]. Gentamycin sulfate was dissolved in distilled water (dH_2_O) and fluconazole in dimethyl sulfoxide (DMSO) (CAS number: 67-68-5, Merck KGaA, Darmstadt, Germany) before combining it with the HBSS solution. Instead of recommended 2% fetal bovine serum (FBS) as a growth supplement, 1% bovine serum albumin V (BSA, BioFroxx, neoLab Migge GmbH, Rischerstr. 7–9, D-69123 Heidelberg, Germany) was used as BSA is advantageous over FBS [24]. Finally, the finished VTM was filtered through a polyethersulfone (PES) liquid filter (pore size 0.2 µm; Sartorius Stedim Biotech, Göttingen, Germany). All preparation steps were performed in a sterile biosafety cabinet to ensure the sterility of the final product (Figure 1). Biosafety cabinets were thoroughly wiped with 70% ethanol and UV irradiated before and after use. Before dispensing, VTM from each batch was aseptically sampled for quality control (QC) testing.

A local manufacturer manufactured two mL conical screw-capped cryotubes, and they were sterilized at BRiCM using autoclave and UV irradiation. Next, 1.5 mL VTM was aseptically put into each sterile tube using a peristaltic pump underneath the biosafety cabinet (Figure 1). Before moving to the next step, media was sampled and QC tested to ensure dispensing operational sterility. Batch information containing a batch number (BN), lot number (LN), manufacturing date (Mfg), and expiration date (Exp) was printed on each tube to assure traceability. A new lot number was assigned for each day’s VTM production. The transport media was supplied in a sealed Ziploc bag with two sterile nylon floc swab sticks (Singuway Biotech Inc, Pingshan, Shenzhen, China), one sterile wooden tongue depressor (Shanghai Sun-Shore Medical Instruments Co. Ltd., Shanghai, China), and an instruction manual for oro-nosal specimen collection as per US-CDC [25]. Each VTM kit was marked with product and batch information and stored at 4 °C until used.

### 2.2. Quality Control of Prepared VTM

To ensure the quality and reliability of the VTM, in-processes and finished product QC is necessary. Ten kits were randomly sampled from each lot for in-house QC checking. Clinical and Laboratory Standard Institute [26] guidelines for “Quality Control of Microbiological Transport Systems- Approved Standard” were adopted for QC checking during and after the VTM production.

#### 2.2.1. Visual Inspection of the VTM Solution

Each sampled VTM kit was examined for sealing, batch information, appropriate content VTM vial integrity, and sealing. Each sampled VTM vial was checked for the right solution volume, odors, color, and pH. In addition, the National Polio & Measles Laboratory, Institute of Public Health (NPML-IPH), under the Director-General of Health Services (DGHS), also evaluated and confirmed the physical appearance of BRiCM VTM.

#### 2.2.2. Chemical Test of VTM Solution

The pH and HBSS concentration of VTM was monitored after each batch production by testing chloride (Cl¯) ion concentration using the method described by Iwaji et al. [27]. British Pharmacopeia (BP) [28] official monograms were followed for QC purposes. We determine the concentration of magnesium sulfate (MgSO_4_), calcium chloride (CaCl_2_), sodium chloride (NaCl), sodium bicarbonate (NaHCO_3_), potassium chloride (KCl), disodium hydrogen phosphate (Na_2_HPO_4_), potassium dihydrogen phosphate (KH_2_PO_4_), and glucose (C_6_H_12_O_6_) in HBSS. Protein concentration was examined in finished VTM using the Kjeldahl method described in Association of Official Analytical Collaboration (AOAC) method 945.23 [29]. Gentamicin sulfate and amphotericin B were checked in finished VTM using liquid chromatography with tandem mass spectrometry (LC-MS/MS) scans [28].

#### 2.2.3. Microbiological Examination of VTM Solution

Each sampled VTM vial was inspected for defects and microbiologically tested for sterility using modified Clinical and Laboratory Standard Institute (CLSI) guidelines [26]. For APC, the AOAC 990.12 [30] method was used. A total of 100 µL of VTM from each of the ten randomly selected tubes from each lot were plated separately on nutrient agar (M001; HIMEDIA, Mumbai, India) in triplicate and incubated at 37 °C for 24 h. *S. aureus* (ATCC25923; ATCC, University Blvd Manassas, VA, USA) was used as the positive control and uninoculated media as the negative control. For YMC, AOAC 997.02 [31] method was used. Each of the ten randomly chosen tubes from each lot contained 100 l of VTM, then were individually plated in triplicate on Sabouraud dextrose agar (SDA) (M063; HIMEDIA, Mumbai, India) and incubated at 25 °C for 72 h. *A. terreus* var. *aureus* (ATCC16793; ATCC, University Blvd Manassas, VA, USA) was used as the positive control and uninoculated media as the negative control. The colony-forming units (CFU) were counted using a standard colony counter immediately after incubation.

### 2.3. Performance Comparison with Available Commercial VTM

The BRiCM VTM was evaluated through a contract research organization (CRO), the National Institute of Laboratory Medicine and Referral Center, Bangladesh (NILMRC). Before sample collection, they also obtained written informed consent from all subjects and/or their legal guardian(s). NILMRC collected SARS-CoV-2 swabs using BRiCM VTM and government-approved Sansure Biotech VTM (No. 680, Luson Road, Yuelu District, 410205, Changsha, Hunan Province, China). They ran both samples in RT-PCR (QuantStudio 5, Thermo Fisher Scientific, Waltham, MA, USA) to detect SARS-CoV-2 RNA.

The CRO, NILMRC, collected 80 randomized and 70 known (12 positives and 58 negatives) SARS-CoV-2 patients’ oro-nasal swabs in both VTM following US-CDC [25] guidelines to compare the performance of the two VTM. They analyzed cycle threshold (Ct) values obtained for both VTM, expressed as mean ± standard error using Microsoft^®^ Excel^®^ for Microsoft 365 MSO.

### 2.4. The Extent of Detection When the Specimen Is Preserved in Storage Conditions

The COVID-19 pandemic has given rise to challenges such as a shortage of RT-PCR machines, PCR detection kits, and technically skilled personnel. In addition, too many specimens need testing with limited resources. Further, the sample collected in VTM produced according to US-CDC, USA formula and preserved at 4 °C can generate reliable results if tested within 2–3 days as per US-CDC claim. Eighty SARS-CoV-2 positive samples confirmed in the previous performance comparison study were stored at 4 °C and tested at two day intervals from day 0 (zero) to the 14th day using the QuantStudio 5, Thermo Fisher Scientific, USA. Ct values of all samples and *N* gene and *ORF* gene were recorded and visualized as aggregates using Levey-Jennings plot as a graph in GraphPad^®^ Prism^®^ 8.4.3.

### 2.5. Stability Study of BRiCM VTM

Accelerated stability was performed using room temperature (RT = 25 °C) incubation to predict the effect of extended storage at 4 °C on VTM’s physical properties, pH, and sterility. A total of 10 mL of VTM were aliquoted from the lot to be tested in twelve 15 mL falcon tubes and stored in RT for four weeks. Two tubes were checked every seven days for QC parameters, one tube for physical parameters and another for microbiological testing. Four-week observations were recorded and analyzed using the Arrhenius equation [32,33] to project VTM stability at the recommended temperature (4 °C). Arrhenius equation describes the mathematical relation between the rate constant of a chemical reaction, reaction temperature, and the activation energy for the molecule under study. Generally, for every 10 °C rise in temperature, the reaction rate may double or quadruple (dependent upon the free energy of activation), meaning that we should expect two times more degradation when we increase the temperature of a solution by 10 °C. The Arrhenius equation is as follows:

Predicted stability = Accelerated stability × 2ΔT/10(1)

Here, ΔT is the difference between the recommended and accelerated study storage temperatures.

## 3. Results

### 3.1. VTM Formulation and Quality Control

During this current COVID-19 pandemic, we intend to develop and manufacture an efficient, cost-effective, and reliable VTM kit within the earliest possible time. While selecting the VTM formula for the production, instead of creating a new recipe from scratch or reverse-engineering an existing commercial recipe, the US-CDC published formulation was adopted [22] with suitable adjustments to attain our goal quickly. The QC results of the manufactured VTM solution are as follows:

#### 3.1.1. Visual Inspection of the VTM Solution

The VTM solution vials were free from mechanical defects and contained 1.5 mL of odorless, clear VTM solution. The National Polio & Measles Laboratory, Institute of Public Health (NPML-IPH) also evaluated and confirmed the physical appearance of the BRiCM VTM solution to be a transparent liquid with no mechanical defects in the vial.

#### 3.1.2. Chemical Tests of VTM Solution

**Figure 2:**
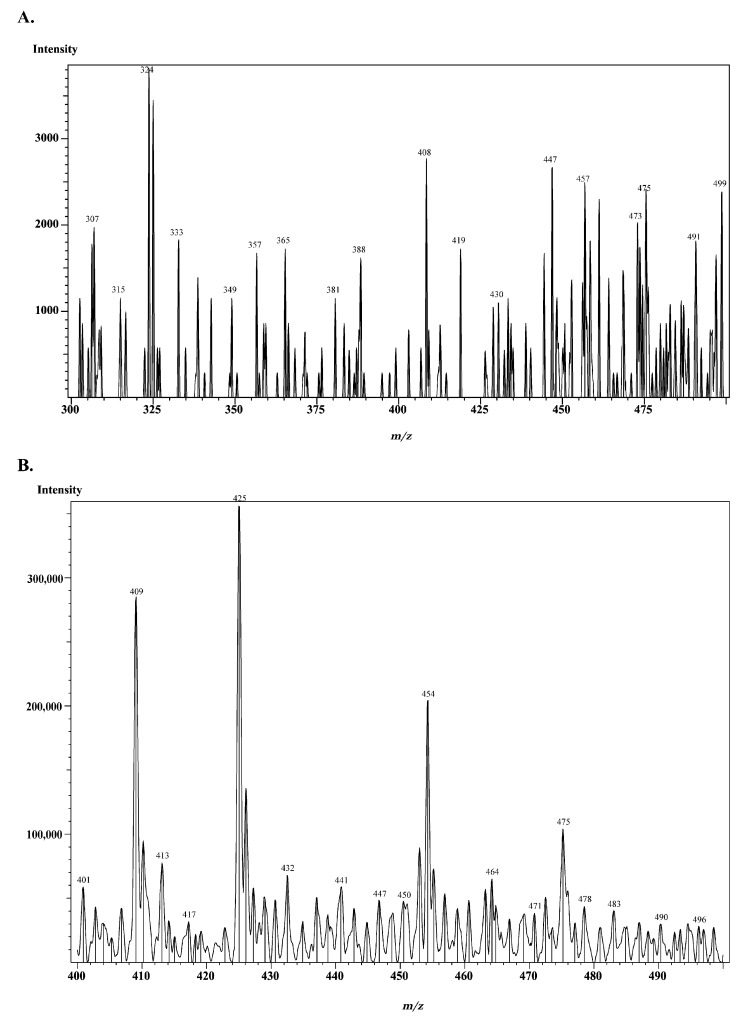
LC-MS scan of VTM. LC-MS scan of VTM for fluconazole (**A**) and gentamicin sulfate (**B**) identification.

The pH was measured during the QC check, and it lay between 6.86 and 6.93. The routine testing of the VTM ingredients (e.g., magnesium sulfate (MgSO_4_), calcium chloride (CaCl_2_), sodium chloride (NaCl), sodium bicarbonate (NaHCO_3_), potassium chloride (KCl), disodium hydrogen phosphate (Na_2_HPO_4_), potassium dihydrogen phosphate (KH_2_PO_4_), glucose (C_6_H_12_O_6_), protein (Table 1), and antibiotics (Figure 2)) was all within the specified range of the US-CDC.

#### 3.1.3. Microbiological Inspection of VTM Solution

No CFU in any plate was observed from either the APC or YMC during a routine QC check and stability study (Supplementary Table S2). The antibacterial and antifungal confirmed by LC-MS/MS scanning (Figure 2) ensures that the VTM can inhibit unwanted microbial growth. In addition, during the stability study, the VTM samples were visually tested for turbidity caused by microbial growth. This confirmed the sterility of the VTM production and packing process.

### 3.2. Performance Comparison

A comparative study between the BRiCM VTM and globally accepted and widely used Sansure Biotech VTM was performed by NILMRC. As a first step, they studied 70 known samples of SARS-CoV-2 stored in both the BRiCM and Sansure VTM. In the case of the Sansure VTM, 12 were found to be positive and 58 negative. The result was the same in the case of the BRiCM VTM, i.e., 12 positive and 58 negative for the same sample (Table 2). As a second step, the oro-nasal swab samples of 80 random COVID-19 symptomatic patients were stored in both the BRiCM and Sansure VTM. In this study, 15 samples (18.75%) were positive, and 65 (81.25%) were negative (Table 3).

In the case of the 70 known specimens, the study shows that 12 known positives were positive, and 58 known negatives were negative in both VTM. The sensitivity and specificity of both VTM were 100% for the known samples. The mean Ct values for the *N* and *ORF* genes of the swabs stored in Sansure Biotech VTM were 31.51 ± 2.18 and 34.05 ± 3.45, respectively. In the case of the BRiCM VTM, the average Ct values for the *N* and *ORF* genes were 32.6 ± 2.11 and 36.11 ± 2.3, respectively. In the *t*-test between the Sansure Biotech VTM and BRiCM VTM for the *N* and the *ORF* genes of the known samples, the *p* values were 0.026 (*p* < 0.05) and 0.1 (*p* > 0.05), respectively. There was a significant difference in the Ct values of the *N* gene and no significant difference in the Ct values of the *ORF* gene between the Sansure Biotech VTM and BRiCM VTM. In the *t*-test between the Sansure Biotech VTM and BRiCM VTM for the *N* and the *ORF* genes of the 80 randomized samples, 15 were positive, 65 were negative, and the *p* values were 0.072 (*p* > 0.05) and 0.052 (*p* > 0.05), respectively. There was no significant difference in the Ct values of the *N* and *ORF* genes at a significance level of 0.05.

### 3.3. Extent of Detection

**Figure 3:**
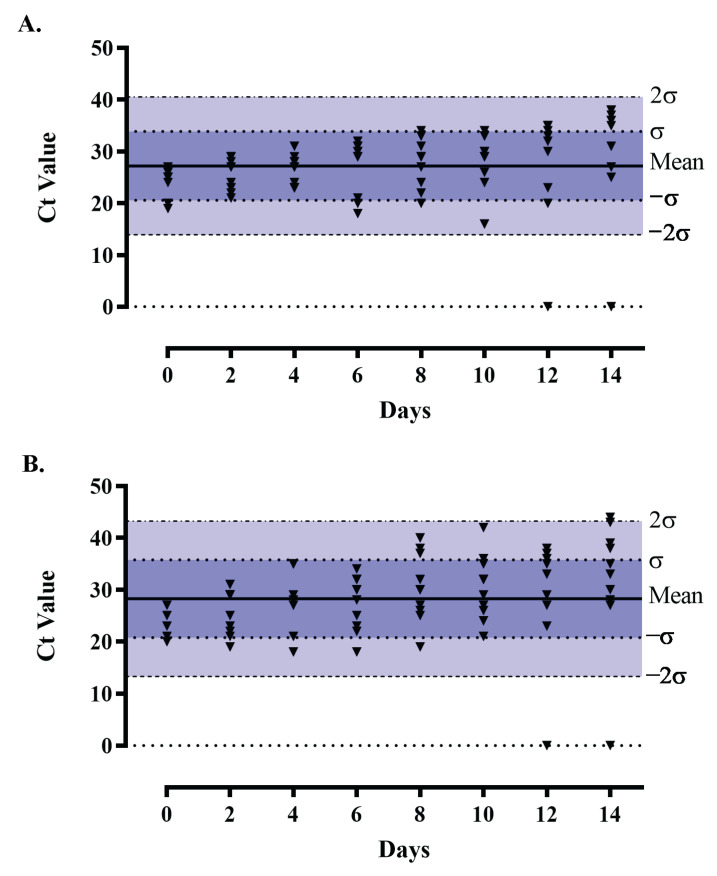
Extent of SARS-CoV-2 RNA detection over 14 days. Ct values of *N* gene (**A**) and *ORF* gene (**B**) amplification from the delayed nucleic acid amplification test (NAAT) plotted in the Levey-Jennings plot. The test days with more than one data point represent the same batch of VTM evaluated for multiple samples. Other than two specimens, deviation in Ct values for both viral RNA and control gene amplification was within the limit of variation, i.e., 2σ.

So far, the literature on how long SARS-CoV-2 RNA remains intact in the storage media is inadequate. In our study, relating to storage period-dependent *N* and *ORF* gene amplification, it is observed that in most of the samples of the said gene amplification, the upper limit of deviation in Ct values within 10 days was below 2. Therefore, the storage period or VTM within 10 days (Figure 3) does not influence PCR detectability. Rogers et al. [34] also evaluated different storage temperatures (18 to 26 °C, 2 to 8 °C, and −10 to −30 °C) of high-titer SARS-CoV-2 specimen-spiked VTM for 14 days and found a mean Ct difference of <3. Aerosolized SARS-CoV-2 can produce culturable SARS-CoV-2 from copper, cardboard, and plastic or stainless-steel surfaces for up to 3–4 h, 24 h, and 2–3 days, respectively [35]. Given its persistence, it is not surprising that SARS-CoV-2 RNA can be reliably amplified from viral transport medium after relatively long storage times, even at room temperature [34].

### 3.4. Stability Study

A four-week-long accelerated stability study at room temperature, where the sample was tested every seven days, showed no significant change in physical appearance, pH, and sterility of the VTM (Supplementary Table S2). The VTM was free from contaminants and remained stable for four weeks under accelerated conditions (Supplementary Table S2). As the VTM remains stable for four weeks at room temperature, according to the Arrhenius equation, it would remain stable for at least four months under the recommended (4 °C) storage conditions.

## 4. Discussion

A reliable, validated, and cost-effective VTM has been developed for the appropriate storage of oropharyngeal and nasopharyngeal specimens of COVID-19 patients following the formula of the US-CDC, USA, tailored to the local requirement, techno-economic situation, and capacity with necessary improvisation. During the current COVID-19 pandemic, many attempts were noticed to produce VTM in variations using the US-CDC SOP DSR-052-05. For example, Smith, Cheng, Chopelas, DuBois-Coyne, Mezghani, Rodriguez, Talay, and Kirby [17] used phenol red (10 mg L^−1^) as a pH indicator to confirm the proper washing of dispense tubing as they used bleach sterilization. However, we used autoclave and UV irradiation to sterilize the VTM tubes, so we followed the US-CDC recommendations not to use any pH indicators. In the BRiCM VTM, 1% BSA instead of the recommended 2% FBS was used. FBS provides essential components for cell survival and proliferation in media, such as hormones, transport proteins, and growth factors, while being low in immunoglobulin (antibodies) content [36]. BSA is an essential component of FBS that provides cryopreservation and anti-adsorption properties that help the virus remain intact in solution rather than breaking down or sticking to plastic [24]. Instead, BSA provides better cryopreservation and gene expression in embryo culture than FBS [24]. We used fluconazole instead of amphotericin B as the antifungal agent because both possess fungicidal properties [23]. In addition, the acceptable quality of amphotericin B was unavailable in the local market during the worldwide pandemic lockdown. The microbiological inspection (Section 3.1.3) shows that 100 µg/mL of fluconazole adequately kept the VTM solution sterile during production and storage.

While checking the pH, we found that the BRiCM VTM pH was between 6.86 and 6.93. The nasopharynx pH for the average individual ranges between 6.10 and 7.92 [37]. Although we found significant difference in the PCR detection of the *N* gene (Section 2.2) between the BRiCM VTM and Sansure Biotech VTM for the known samples, this was probably due to the consecutive sampling of the specimen from an individual in the Sansure VTM and then in the BRiCM VTM, resulting in a low viral load in the later swab to begin with. However, it was still within the recommended Ct value (<40) [34]. For the *ORF* gene detection of the known samples and the *N* and *ORF* genes of the unknown samples, there was no significant difference between the Sansure Biotech and BRiCM VTM stored samples at a significance level of 0.05.

For sterility checking of the VTM, the US-CDC [22] SOP (DSR-052-02) recommended only a blood agar plate or equivalent. Blood agar can detect a wide range of hemolytic bacteria and yeasts. However, if yeast and bacteria are simultaneously present in the sample, bacteria may inhibit the growth of the yeast [38]. So, the AOAC 990.12 [30] method for aerobic plate count (APC) and AOAC 997.02 [31] method for yeast and mould count (YMC) was used to confirm the sterility. However, Smith, Cheng, Chopelas, DuBois-Coyne, Mezghani, Rodriguez, Talay, and Kirby [17] proved their VTM’s antimicrobial stability only by a killing study using *Escherichia coli* and *Candida albicans*. We used *Staphylococcus aureus* in APC and *Aspergillus terreus* var. *aureus* in YMC as the positive control during the sterility tests.

In a comparison study of VTM and swabs, Garnett et al. [39] reported that transport media such as Dulbecco’s Modified Eagle Medium (DMEM), phosphate buffered saline (PBS), 100% ethanol, 0.9% normal saline, and VTM do not significantly influence the PCR detectability of the viral specimen. Rogers, Baumann, Borillo, Kagan, Batterman, Galdzicka, Marlowe, and McAdam [34] also found no significant difference between the Ct values of UTM, UTM-RT, ESwab, M4, and saline (0.9% NaCl) stored NP and OP swabs. Druce et al. [40] also reported a similar finding in their comparison study. According to Wang, Hu, Hu, Zhu, Liu, Zhang, Wang, Xiang, Cheng, Xiong, Zhao, Li, Wang, and Peng [21], a Ct value less than 37 is a positive test result, a Ct-value of 40 or more is negative, and a Ct-value of 37 to 40 requires confirmation by retesting.

During the stability study, the VTM was free from contaminants and remained stable for four weeks under accelerated conditions (Supplementary Table S2). As the VTM remains stable for four weeks at room temperature, according to the Arrhenius equation, it would remain stable for at least four months under the recommended (4 °C) storage conditions. The pH study showed no significant (*p* = 0.05) difference. In addition, according to the US-CDC [22] SOP (DSR-052-02), a VTM stored at 4 °C has a shelf life of one year. In an accelerated (2 weeks at 56 °C) stability study, Smith, Cheng, Chopelas, DuBois-Coyne, Mezghani, Rodriguez, Talay, and Kirby [17] projected their formulated VTM would be stable for up to 4 months at room temperature. However, they didn’t report any extent of the detection study to indicate whether their VTM-containing specimen remains stable at room temperature or not. In our extent of detection study, we found that for most of the BRiCM VTM-spiked sample, the upper limit of deviation in the Ct value was within 2 during the first 10 days. Therefore, it is safe to conclude that BRiCM stored specimens can be reliably tested within 10 days.

## 5. Conclusions

This article describes our effort to develop the VTM for assembling a COVID-19 specimen collection kit locally in the face of the scarcity of imported diagnostic tools and reagents due to the international lockdown and commercial embargo caused as a result of the SARS-CoV-2 pandemic. From the VTM formulation, production, and evaluation up to quality control and validation procedures, both the in-house and independent CRO have been described. It is evident from the relevant studies that the VTM remains stable for up to four months and can effectively store viral specimens under recommended conditions for at least four weeks. In addition, its performance is similar to other commercially available and government-approved VTM solutions. It is compatible with existing RNA extraction and RT-PCR amplification methods, reagents, and tools. The delayed RT-PCR test of the COVID-19 specimen stored in the VTM showed no significant influence on viral RNA detection. Most of the positive samples in the performance comparison and comprehensive study showed a Ct value of around 30, meaning delayed detection may impact lower titer. The development and production of this VTM have paved the way to overcome the scarcity of diagnostic tools and reagents for COVID-19 tests in high volume. This work is expected to inspire others to produce necessary medical devices in Bangladesh.

## Figures and Tables

**Table 1:** Chemical analysis of BRiCM VTM finished product.

Parameters	Formulation	Finished Product Test Result
Protein	1.0%	0.98%
Sodium Chloride (NaCl)	0.8%	0.8%
Glucose (C_6_H_12_O_6_)	0.1%	0.1%
Potassium Chloride (KCl)	0.04%	0.04%
Sodium Bicarbonate (NaHCO_3_)	0.04%	0.03%
Potassium Dihydrogen Phosphate (KH_2_PO_4_)	0.01%	0.02% (as PO_4_)
Disodium Hydrogen Phosphate (Na_2_HPO_4_)	0.01%	
Magnesium Sulphate (MgSO_4_)	0.01%	0.01%
Calcium Chloride (CaCl_2_)	0.01%	0.01%
Antibiotic	Gentamycin and Fluconazole	Confirmed

**Table 2:** Ct values of *N* and *ORF* gene amplification for the stored known specimens in Sansure Biotech VTM and BRiCM VTM.

ID	*N* Gene		*ORF* Gene	
	Sansure Biotech VTM	BRiCM VTM	Sansure Biotech VTM	BRiCM VTM
K 01	31.92	32.12	38.45	38.88
K 02	29.78	30.34	31.29	32.45
K 03	32.35	33.56	37.19	37.54
K 04	29.12	30.45	30.23	34.67
K 05	28.23	29.21	34.23	35.34
K 06	30.12	31.45	34.32	36.47
K 07	32.21	33.45	37.28	38.02
K 08	34.12	35.15	27.34	31.49
K 09	33.39	34.54	33.32	36.98
K 10	33.67	34.56	35.88	37.02
K 11	34.25	35.41	37.65	38.49
K 12	28.91	30.96	31.45	35.96

**Table 3:** Ct values of *N* and *ORF* gene amplification for the stored randomized specimens in Sansure Biotech VTM and BRiCM VTM.

ID	*N* Gene		*ORF* Gene	
	Sansure Biotech VTM	BRiCM VTM	Sansure Biotech VTM	BRiCM VTM
R 03	29.69	29.72	29.7	30.28
R 05	30.25	30.78	0	0
R 06	31.95	32.12	32.53	33.37
R 09	31.27	31.29	31.83	36.34
R 17	31.63	31.54	33.81	33.87
R 25	29.53	29.56	32.14	32.32
R 33	30.67	32.87	34.15	34.45
R 40	34.67	34.88	30.9	31.45
R 43	30.52	30.56	35.19	35.32
R 51	29.63	29.78	31.5	31.92
R 56	32.39	32.53	37.05	37.19
R 59	31.54	31.82	0	0
R 70	30.13	30.32	36.32	36.54
R 73	34.29	34.45	35.72	37.38
R 78	32.69	32.78	36.85	37.45

## Data Availability

We summarized the datasets used in this manuscript and presented them as supporting information for publication. The corresponding author will make any other relevant information available upon reasonable request.

## Supplementary Materials

The following supporting information can be downloaded at: https://www.mdpi.com/article/10.3390/diagnostics13091622/s1, Table S1: Stability study design of BRiCM VTM; Table S2: Stability study result of BRiCM VTM; Figure S1: Technical evaluation for “Corona virus swab specimen collection kit”.
