# Supplementary material for: Performance Evaluation of SARS-CoV-2 Viral Transport Medium Produced by Bangladesh Reference Institute for Chemical Measurements
Source: Diagnostics (Basel). 2023 May 4;13(9):1622. doi: 10.3390/diagnostics13091622 (PMC10177798; doi:10.3390/diagnostics13091622)
Supplement: Supplementary file 1 [file diagnostics-13-01622-s001.zip › diagnostics-2183834-supplementary.pdf]

Supplementary Table S1: Stability study design of BRiCM VTM

If VTM is stable at 25°C	Then predicted shelf life at recommended 4°C*
Week 0	0 Month
Week 1	1 Month
Week 2	2 Month
Week 3	3 Month
Week 4	4 Month

* Accelerated stability testing was done according to the following formula derived from the Arrhenius equation; Here, ΔT is the difference between the recommended storage temperature and the accelerated study storage temperature.

Supplementary Table S2: Stability study result of BRiCM VTM

Date	Tube No.*	Test Repeat	Parameters				
			pH	Significant difference	Temperature	Total Microbial Count	Yeast and Mold
29/09/2021	1	1	6.69		24.6 °C		
		2	6.73		24.7 °C		
		3	6.74		24.8 °C		
		4	6.74		24.8 °C		
		5	6.74		24.8 °C		
	2					Nil	Nil
6/10/2021	1	1	6.73	0.052	25.7 °C		
		2	6.71		25.7 °C		
		3	6.68		25.7 °C		
		4	6.68		25.7 °C		
		5	6.66		25.7 °C		
	2					Nil	Nil
13/10/2021	1	1	6.91	0.000	24.3 °C		
		2	6.91		24.3 °C		
		3	6.86		24.2 °C		
		4	6.86		24.3 °C		
		5	6.86		24.4 °C		
	2					Nil	Nil
20/10/2021	1	1	6.91	0.000	25.4 °C		
		2	6.91		25.4 °C		
		3	6.90		25.4 °C		
		4	6.89		25.4 °C		
		5	6.89		25.5 °C		
	2					Nil	Nil
27/10/2021	1	1	6.97	0.000	24.2 °C		
		2	6.95		24.3 °C		
		3	6.93		24.3 °C		
		4	6.93		24.3 °C		
		5	6.93		24.3 °C		
	2					Nil	Nil

* For each of the data point, two tubes were checked, one tube for physical parameters and another for microbiological testing.

Figure S1: Technical evaluation for Coronavirus swab specimen collection kit

Government of the People's Republic of Bangladesh
Ministry of Health and Family Welfare
Directorate General of Health Services
Mohakhali, Dhaka 1212

Memo: ADG (Admin)/ DGHS/2020/July   Dated: 09.07.2020

Technical Evaluation for "Corona virus Swab specimen collection Kit" for DGHS, MOHFW, Bangladesh.

Due to COVID-19 pandemic crisis situation, requirement of huge sample collection kits and unavailability of imported VTM/sample collection kit, DGHS has planned to get an alternative source from local company. In this regard, Bangladesh Council of Scientific and Industrial Research (BCSIR) submitted some kits to DGHS with a letter, memo no. 39.02.0000.041.18.20/877 signed by additional secretary Mr. Farul Ahmed, Chairman, BCSIR. .

For kit evaluation, ADG-Admin, DGHS has sent some **Corona Virus specimen collection kits** (photo attached) to Virologist, National Polio and Measles Laboratory(NPML-IPH) of Institute of Public Health(IPH), Dhaka.

With guidance of ADG, Admin, DGHS, the Virologist, National Polio and Measles laboratory (NPML), Institute of Public Health has conducted following evaluation activities.

Evaluation steps:

A. **Physical evaluation:** Transparent kit with poly package named Corona virus specimen collection kits, contains following:

i. A 5 ml, violet capped plastic tube contains "3 ml Transparent liquid labeled with Viral Transport Medium", PN: 132022; BN: 20200503; LN: 03052020
ii. Two sterile swab(cotton) sticks in a sealed poly pack
iii. One Sterile Tongue depressor in a sealed poly pack
iv. Instruction manual

- MFG date: May 2020; Exp. date: April 2021
- Sealed as sterile(as per company)
- Store: at 2-8 degree C; Do not Freeze
- Labeled as a product of DRICM(Designated reference institute for Chemical Measurements), Bangladesh.

B. laboratory evaluation activities for sample collection kits:

1. DGHS Sample collection team collected Nasal-Throat swab samples from suspected COVID-19 cases, using these kits. The sample tubes containing liquid media were sent to IPH in cool box with ice packs.
2. COVID-19 lab, NPML-IPH received labeled tubes with swab-samples from DGHS sample collectors. :NO damage found to swabs/sticks in tubes with liquid VTM. VTM was found clear.
3. Received samples in VTM were processed and tested for SARS-CoV-2 by real-time PCR.
4. Real-time RT-PCR Test for collected samples by these DRICM kits, able to detect SARS-CoV-2 RNA in some samples.

[illegible]
9|7|2020   P-1

Continue

Inference: rRT-PCR test (by sansure kit supplied by DGHS) has detected SARS-CoV-2 virus RNA in some throat and nasal swab samples collected by supplied "sterile Corona Virus specimen collection kit".

Note: The capability of DRICM VTM to keep virus alive is not tested, for which "virus/sample in VTM" was needed to grow(virus isolation) in cell culture facilities. NPML did not do virus isolation test.

The kit evaluation by:
9/7/2020
Dr. Khondoker Mahbuba Jamil
Virologist and lead
CORONA lab &
BSL2 National Polio & Measles Laboratory
Institute of Public Health (IPH)
MOHFW, Bangladesh.
email: mahbuba45@hotmail.com; Dated: 09.7.2020

Reviewed by:
09.07.20
Prof. Dr. Nasima Sultana
Additional Director-General (Admin)
DGHS, MOHFW, Bangladesh
Dated: 09.7.2020
Email: adgadmin@ld.dghs.gov.bd
